# Supplementary material for: Validation Study for Non-Invasive Prediction of IDH Mutation Status in Patients with Glioma Using In Vivo 1H-Magnetic Resonance Spectroscopy and Machine Learning
Source: Cancers (Basel). 2022 Jun 2;14(11):2762. doi: 10.3390/cancers14112762 (PMC9179368; doi:10.3390/cancers14112762)
Supplement: Supplementary file 1 [file cancers-14-02762-s001.zip › cancers-1729603-supplementary.pdf]

cancers

MDPI

Supplementary materials

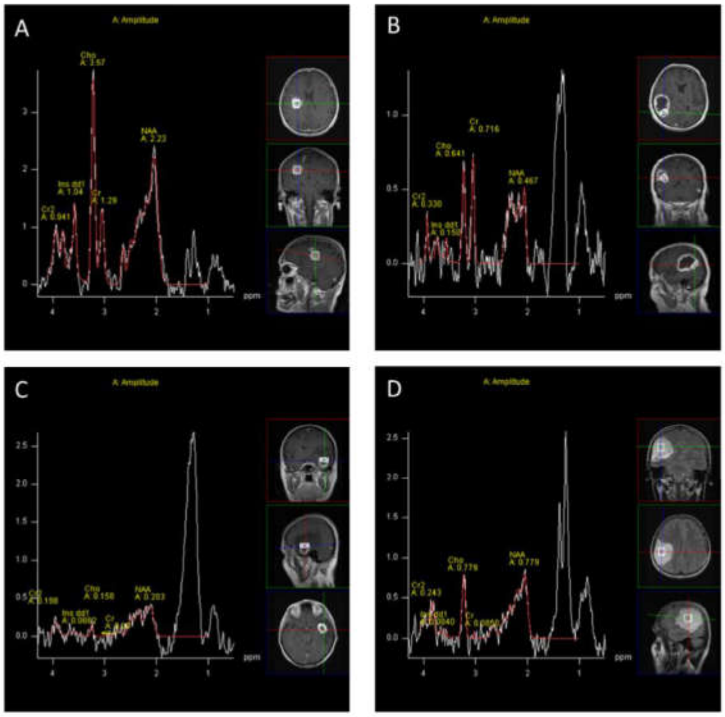

Figure S1: Quality of ^{1}H-MR spectra

Spectra shown in (A) and (B) fulfill the inclusion criteria (amplitude Cho > 0.2, Cr > 0.1, M-Ins > 0, Cr2 > 0), spectra in (C) and (D) were excluded. The amplitudes of the metabolites were calculated by fitting the peaks within the vendor-specific „Spectroscopy" card and are given in arbitrary units. Their absolute value depends on several technical parameters, e.g. magnetic field strength, receiver coil and measurement parameters.

A) Relatively high amplitudes of all metabolites, voxel size: 18 mm x 16 mm x 20 mm.
B) Relatively low amplitudes of all metabolites, voxel adapted to the size and shape of the tumor (necrotic portions of the tumor are mainly excluded); voxel size: 12 mm x 12 mm x 18 mm.
C) Very low amplitdes of relevant metabolites, excluded because of too low amplitudes of Cho (0.158) and Cr (0); voxel size: 18 mm x 17 mm x 10 mm.
D) Very low amplitudes of relevant metabolites, excluded because of too low amplitude of Cr (0.085); voxel size: 18 mm x 16 mm x 18 mm.

Figure S1. Quality of ^{1}H-MR spectra.

Table S1. Technical parameters and conditions for the original and validation cohort.

	Original Cohort	Validation Cohort
Time period for data acquisition	2015 - 2019	2002 - 2007
Scanner field strength	3.0 T	1.5 T
Coil used for signal reception	20-channel phased-array head/neck coil	Single channel head coil
Sequence for single voxel spectroscopy	PRESS	PRESS
Repetition time (TR)	2000 ms	1500 ms
Echo time (TE)	30 ms	30 ms
Number of acquisitions averaged	100	128
Spectral width	1200 Hz	1000 Hz
Data points	1024	1024
Voxel size	Constant (14 mm x 14 mm x 14 mm)	Variable (min.: 10 mm x 10 mm x 10 mm, max.: 20 mm x 20 mm x 20 mm), adapted to tumor size and shape
Voxel positioning guided by	Standard MRI (T2-weighted TSE, T2-weighed FLAIR, T1-weighted SE post contrast) ^{18}F-FET PET	Standard MRI (T2-weighted TSE, T2-weighed FLAIR, T1-weighted SE post contrast)

FLAIR: Fluid Attenuated Inversion Recovery; PRESS: Point RESolved Spectroscopy; SE: Spin Echo; TSE: Turbo Spin Echo.

Text S1. Matching of the original and the validation cohort.

The classification algorithm was trained on the original cohort to identify a set of predictive features, i.e., a set of predictive bins (bin size 4 data points). For application of the trained model to the validation cohort, it must be ensured that the corresponding bins in the validation cohort are unambiguously identified. To this end, an optimal matching of the spectral data of both cohorts is required. For the training data, 1024 data points were collected over a spectral width of 1200 Hz and at a field strength of 3.0 T, while for the validation data 1024 data points were collected in a spectral width of 1000 Hz and at a field strength of 1.5 T. In magnetic resonance spectroscopy, field strength and resonance frequency are directly proportional to each other. Therefore, two metabolite signals that are separated by 200 Hz at 3.0 T will be separated by 100 Hz at 1.5 T. Consequently, to obtain an optimal matching of features between different data sets, differences in field strength, spectral width and number of acquired data points were considered. Empty data points resulting from this adjustment were imputed using the mean of adjacent data points.

Text S2. Workflow of data collection and analysis in the original and validation cohort.

In both the original and the validation cohort, spectra were aligned with respect to the easily identifiable choline signal, followed by normalization to total spectral area and $\log_2$ transformation. To ensure meaningful total spectral area and logarithm results, we did not allow negative values and set a lower bound of 1 for all values prior to normalization. Furthermore, as explained in Supplement Text S1 spectra of the validation cohort were matched to the original cohort. Next, all spectra of the original cohort were used for training of the SVM based classification approach. The trained model was then applied without any further modification to the preprocessed spectra of the validation cohort and results were recorded.

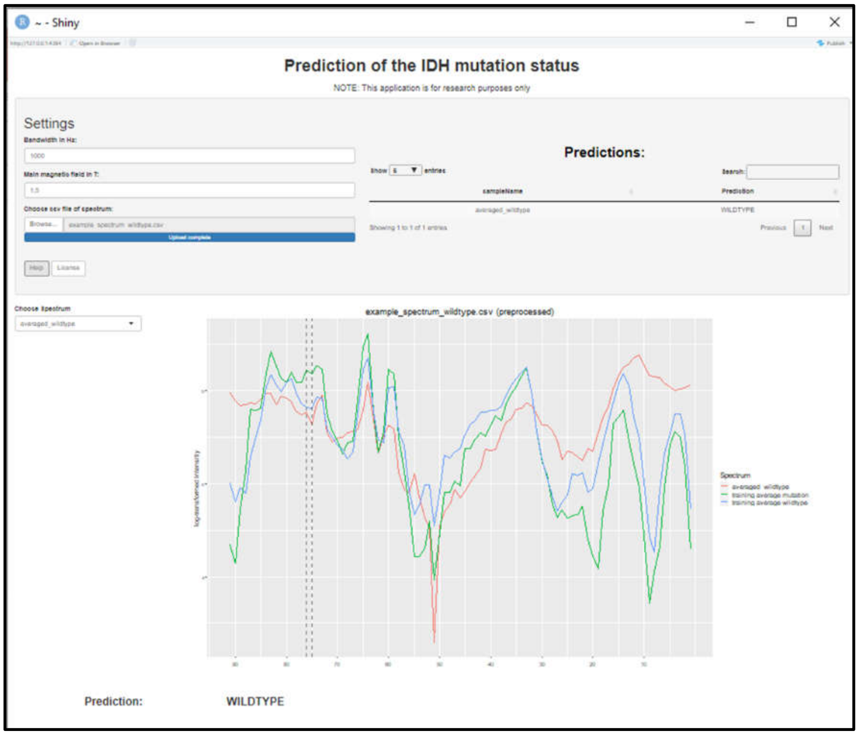

Figure S2. Screenshot of our App predicting *IDH* mutation status. The app is available at https://www.uni-regensburg.de/medicine/functional-genomics/staff/prof-wolfram-gronwald/software/index.html or as a web application: https://IDH-prediction.spang-lab.de.

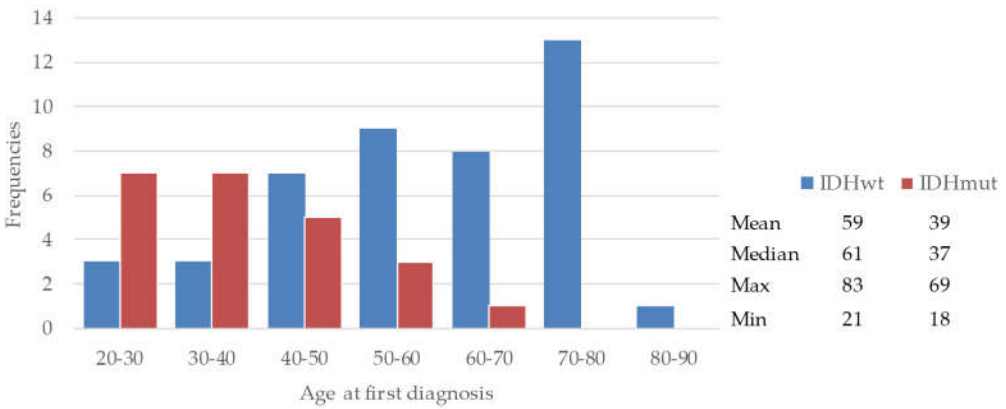

Figure S3. Patient characteristics: Distribution of age at first diagnosis in the *IDH*wt group (n = 44) and the *IDH*mut group (n = 23). *IDH*: *isocitrate dehydrogenase*; mut: mutated; wt: wildtype.

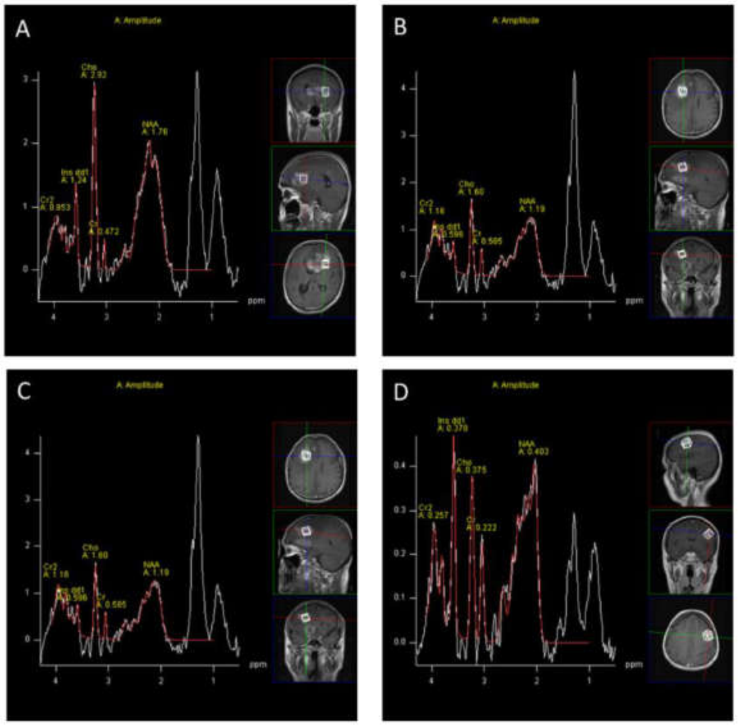

Figure S4: Examples of false positive and false negative predicted ^{1}H-MR spectra

All spectra shown in (A) - (D) fulfill the inclusion criteria (amplitude Cho > 0.2, Cr > 0.1, M-Ins > 0, Cr2 > 0).

(A) and (B) histologically confirmed *IDH*mut status (sequencing), false negative predicted (*IDH*wt) in analysis of ^{1}H-MR spectra.

(C) and (D) histologically confirmed *IDH*wt status (sequencing), false positive predicted (*IDH*mut) in analysis of ^{1}H-MR spectra.

Figure S4. Examples of false positive and false negative predicted ^{1}H-MR spectra.
